# Comparison of robot-assisted thoracic surgery versus video-assisted thoracic surgery in the treatment of lung cancer: a systematic review and meta-analysis of prospective studies

**DOI:** 10.3389/fonc.2023.1271709

**Published:** 2023-10-30

**Authors:** Shibo Huang, Xiaolong Huang, Zhilong Huang, Raoshan Luo, Weiming Liang

**Affiliations:** The First Affiliated Hospital of Guangxi University of Science and Technology, Guangxi University of Science and Technology, Liuzhou, Guangxi, China

**Keywords:** robot-assisted thoracic surgery_1_, video-assisted thoracic surgery_2_, lung cancer_3_, non-small cell lung cancer_4_, complication_5_

## Abstract

**Introduction:**

Previous studies have compared robot-assisted thoracic surgery(RATS) with video-assisted thoracic surgery (VATS) in the treatment of patients with lung cancer, but results were conflicting. The present meta-analysis aimed to compare the clinical outcomes of RATS with VATS in the treatment of patients with lung cancer.

**Materials and methods:**

Web of Science, PubMed, Cochrane Library and Embase were comprehensively searched for randomized controlled trials or prospective cohort studies comparing the clinical outcomes of RATS and VATS from inception to 22 July 2023. The Cochrane Risk of Bias tool was used to assess risk of bias. Meta-analyses of length of hospital stay, postoperative duration of drainage, postoperative complications, operative time, conversion, estimated blood loss, the number of dissected lymph nodes and stations, 30-day readmission and 30-day mortality were performed.

**Results:**

In total 5 studies were included in the meta-analysis. A total of 614 patients were included, of which 299 patients were treated by RATS and 315 patients treated by VATS. Blood loss was significantly less in RATS group than that in VATS (MD = −17.14, 95% CI −29.96 ~ −4.33, P = 0.009). More nodes stations were dissected in RATS group compared with VATS group(MD= 1.07, 95% CI 0.79 ~ 1.36, P < 0.001). No significant difference occurred between RATS and VATS in length of hospital stay(MD= −0.19, 95% CI −0.98~0.61), readmission(OR=0.74, 95%CI 0.36~1.51, P=0.41), operative time(MD=11.43 95% CI −8.41~31.26, P=0.26), conversion(OR=0.58, 95% CI 0.29~1.17, P=0.13), number of dissected lymph nodes(MD=0.98, 95% CI −0.02~1.97, P=0.05), upstaging rate(OR =0.67, 95% CI 0.38 ~ 1.18, P =0.16, I^2 =^ 0%), time of chest tube drainage (MD= −0.34, 95%CI −0.84~0.15, P=0.17), post-operative complications(OR=0.76, 95% CI 0.52~ 1.11, P=0.16) and total cost(MD = 3103.48, 95% CI −575.78 ~ 6782.74, P=0.1, I^2 =^ 99%).

**Conclusion:**

RATS is a feasible and safe treatment that can achieve better surgical outcomes compared with VATS in terms of short-term outcomes. Except of higher total cost, RATS has obvious advantage in lymphadenectomy and control of intraoperative bleeding. However, large sample and long follow-up randomized clinical trials comparing RATS with VATS are still necessary to better demonstrate the advantages of RATS for lung cancer.

**Systematic review registration:**

https://www.crd.york.ac.uk/prospero/, Identifier CRD42023446653.

## 1 Introduction

Lung cancer is still the most common malignancy worldwide which seriously threaten human health and life, accounting for 11.4% of all cancer cases and 18% of all deaths due to cancer (1–3). Lung cancer has two subtypes: small cell lung cancer which account for 15% and non-small cell lung cancer (NSCLC) which account for 85% (4). The preferred treatment for NSCLC is surgical resection. Thought the traditional open surgery approach is effective, it has been shown to be associated with substantial postoperative complications and mortality (5). VATS has been widely used in thoracic surgery worldwide which could maintain similar long-term outcomes and obviously improve short-term outcomes compared with open thoracotomy (6, 7). However, VATS has several limitations, including of difficult hand-eye coordination, a long learning curve, lack of flexibility, and the disadvantage in terms of mediastinal lymphadenectomy, which may restrict its development (8–10). Since the first robot-assisted thoracic surgery(RATS) performed in 2003, RATS has developed quickly into a relatively new platform for surgical resection, which has been considered as an alternative to VATS (11). RATS seems to have some advantages over VATS, including of high definition three-dimensional optics, better ergonomics, shorter learning curve, small-wristed instrument motions, outstanding maneuverability of instruments and better tremor suppression, improving the perioperative outcomes (12–15).

Though previous systematic reviews and meta-analysis have sought to compare operative approaches for lung cancer, their conclusions were conflicting on whether or not it benefits to transitioning to RATS for surgeons who have mastered VATS (16–19). Due to the shortage of strict inclusion criteria, a large amount of low evidence level RATS studies such as retrospective studies, database studies, and even other metaanalysis was included in above studies, which led to duplication of studied patients and resulted in probably unreliable conclusions.

In the present study, strict inclusion criteria was performed and only randomized controlled trials or prospective cohort studies were included to compare outcomes of RATS versus VATS in the treatment of lung cancer. The primary objective of the review was to examine perioperative complications. Secondary outcomes included hospital stay, operation time, intraoperative bleeding, number of dissected lymph nodes stations, number of lymph nodes cleared during surgery, conversion rate during surgery, postoperative thoracic drainage time, postoperative hospital stay, incidence of early postoperative complications, 30-day mortality, 30-day readmission, total cost.

## 2 Materials and methods

### 2.1 Search strategy

Our study has been registered at PROSPERO under registration number CRD42023446653. The systemic review and meta-analysis was completed according to the Preferred Reporting Project for Systematic Review and Meta-Analysis (PRISMA) 2020 guidelines. A systematic literature search for studies investigating RATS versus VATS for lung cancer was conducted in Medline (1946 to July 22, 2023), Embase (1974 to July 22, 2023), Web of Science (1966 to July 22, 2023), and CENTRAL(1995 to July 22, 2023) by two independent investigators, using the following searching terms: “Lung cancer” AND “Robotic” AND “Thoracoscopy” AND (“randomized controlled trial” OR “Prospective Studies”). The details of the searching record in four databases were shown in **Supplement Tables 1**–**4**. The bibliographies of the identified articles including of relevant reviews and meta-analyses were also manually checked to identify additional eligible studies. Besides, we also searched three clinical trial registries (ClinicalTrials.gov, Controlled-trials.com, Umin.ac.jp/ctr/index. The htm) for unpublished clinical studies.

### 2.2 Inclusion and exclusion criteria

Inclusion criteria were as follows: (1) a randomized controlled trial or prospective cohort study comparing RATS with VATS for the treatment of lobectomy or segmentectomy in patients with lung cancer; (2)full-text articles reporting at least one of the following outcomes: perioperative complications, hospital stay, operation time, intraoperative bleeding, number of dissected lymph nodes stations, number of lymph nodes cleared during surgery, conversion rate during surgery, postoperative thoracic drainage time, postoperative hospital stay, incidence of early postoperative complications, 30-day mortality, 30-day readmission, total cost, upstaging rate; (3) if two or more researches included the same cohort, only the latest published one was included.

Literatures meeting the following criteria were excluded: (1)other types of articles, such an reviews, case reports, animal experimental studies, letters to the editor, conference abstracts, comments, database studies; (2) no lung cancer cases; (3)small sample size: less than 10 participants in RATS group; (4)retrospective studies.

### 2.3 Data extraction

Two independent investigators initially extracted relevant data of included studies, and a third reviewer checked it. The following data were extracted: publication year, country, first author, sample size (intervention arm and control arm), study design, surgical techniques, age, sex, site of tumor, TNM stage, the number of dissected lymph nodes, the number of dissected lymph stations, operative time, conversion, estimated blood loss, postoperative duration of drainage, length of hospital stay, postoperative complications, 30-day readmission, 30-day mortality, upstaging rate, total cost.

### 2.4 Risk of bias assessment

The risk of bias in the studies included was assessed by two independent reviewers using the Cochrane Risk of Bias tool, which includes seven domains: (1)random sequence generation; (2)allocation concealment; (3)blinding of participants and personnel; (4)blinding of outcome assessment; (5)incomplete outcome data; (6)selective reporting; (7)others bias. If there were discrepancies, the controversial results were resolved by group discussion.

### 2.5 Data analysis and statistical methods

The selection of studies and duplicate removal were conducted using EndNote (Version 20; Clarivate Analytics). All results of the studies were analyzed using Review Manager 5.3 (Cochrane Collaboration, Oxford, UK). Odds ratio (OR) with 95% confidence interval (CI) were used to compare binary variables. Continuous variables were compared using weighted mean difference (WMD) with a 95% CI. The medians and interquartile ranges of continuous data were converted to means and standard deviations. For all meta-analyses, the Cochrane Q p value and I^2^ statistic were applied to check heterogeneity. Pooled data were analyzed using a fixed-effect model (FEM) if heterogeneity was low or moderate (I^2^ <50%), or a random-effect model(REM) if heterogeneity was high (I^2^ ≥50%). Statistical heterogeneity was assessed using a standard chi-square test and was considered significant at P<0.05. The potential publication bias was evaluated by visually inspecting the funnel plots.

## 3 Results

### 3.1 Literature search

**Figure 1:**
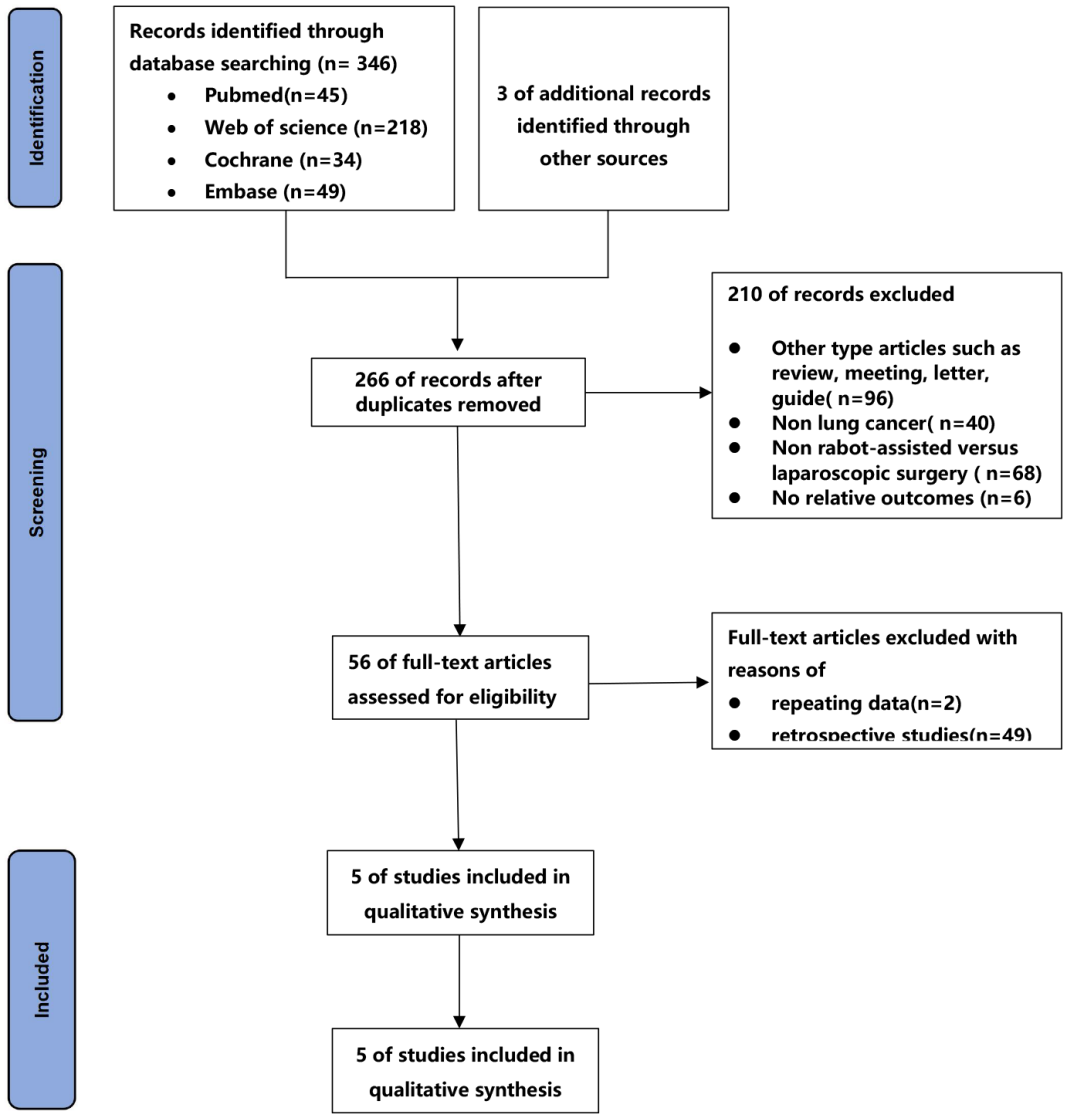
Flow chart of literature search strategies.

The process of the studies selection and inclusion was shown in **Figure 1**. A total of 346 articles were retrieved from four databases, and 3 article was obtained by checking the bibliographies of the identified articles. Finally, a total of 5 prospective studies(20–24) were included in the final meta-analysis based on inclusion and exclusion criteria.

### 3.2 Characteristics of the included studies

In total, 5 studies consist of 614 patients were included in the meta-analysis, of which 299 patients were treated by RATS and 315 patients were treated by VATS. The five studies came from different countries (Korea, France, Brazilian, Italy, China) and were all prospective studies in recent 10 years. The detailed information and baseline characteristics of the included patients is presented in **Table 1**. Three of the studies were prospective randomized controlled trials, and another two were prospective non-randomized controlled studies with the choice between VATS and RATS depending on patient-preference or robot availability.

**Table 1:** Characteristics of the included studies.

study	year	country	design	Study Period	group	cases	mean age	Sex (M/F)	Surgical techniques	Tumor Site (Right/Left)	TNM stage (0/I/II/III,IV)
Park	2017	Korea	P	2011-2013	RATS VATS	12 17	62.60 61.20	7/5 7/10	4 arms	6/6 13/4	0/29/0/0
Gonde	2017	France	P	2014-2015	RATS VATS	57 55	60.65 62.65	31/26 41/14	3 arms	NA	0/52/23/7/1
Terra	2019	Brazilian	P	2015-2017	RATS VATS	37 39	68.40 65.70	17/20 17/22	3 arms	25/12 21/18	NA
Veronesi	2021	Italy	P	2017-2018	RATS VATS	38 39	69.00 69.00	21/17 23/16	NA	24/14 23/16	0/67/5/0
Jin	2022	China	P	2017-2020	RATS VATS	157 163	60.30 60.95	81/76 76/87	3 arms	NA	3/265/25/27

P, Prospective Studies; RATS, robot-assisted thoracic surgery; VATS, video-assisted thoracic surgery; M, male; F, female; NA, not available.

### 3.3 Risk of bias

**Figure 2:**
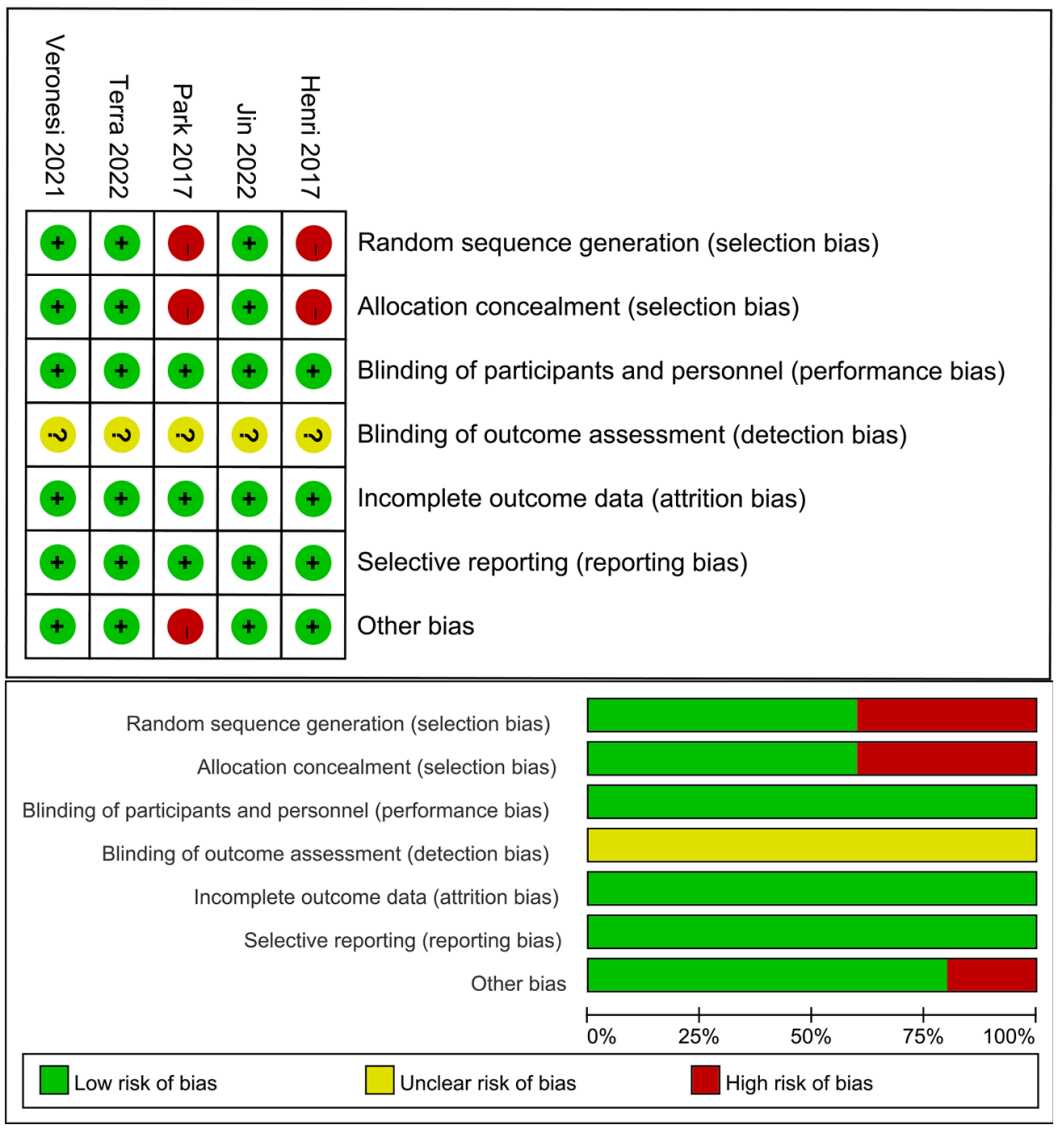
Risk of bias assessment for the included studies.

The assessment of the risk of bias are summarized in **Figure 2**. Among the 5 studies, an adequate randomized sequence was reported in 3 studies, appropriate allocation concealment was generated in 3 studies, the blinding of participants was clear in 5 studies, the blinding of outcome assessors was generated in no studies, outcome data were complete in 5 studies, 5 studies had no selective reporting, and 4 studies had no other bias.

### 3.4 Clinical outcomes

**Figure 3:**
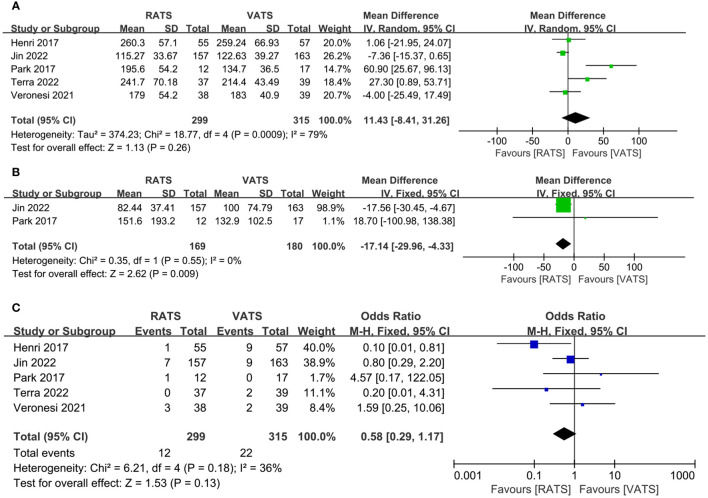
Forest plot of the meta-analysis for intraoperative parameters. **(A)** Operation time. **(B)** Estimated blood loss. **(C)** Conversion.

**Table 2** showed results of meta-analysis for all clinical outcomes. The operative time was reported in 5 literature, and no significant difference occurred between two groups(WMD =11.43, 95% CI −8.41 ~ 31.26, P =0.21, I^2 =^ 79%) (**Figure 3A**). Two studies reported the estimated blood loss. The estimated blood loss in RATS group was significantly lower than that in VATS group(WMD= −17.14, 95% CI −29.96 ~ −4.33, P=0.009, I^2 =^ 0%) (**Figure 3B**). Five studies reported the conversion cases, conversion rate was not statistically significant between two group(WMD=0.58, 95% CI 0.29 ~ 1.17, P =0.13, I^2 =^ 36%) (**Figure 3C**).

**Table 2:** Results of the meta-analysis.

Outcomes	No. of studies	Sample size	Heterogeneity		Overall effect size	95% CI of overall effect	P Value
		RATS VATS	I^2^(%)	P Value			
Operation time (min)	5	299 315	79	<0.001	WMD=11.43	-8.41 ~31.26	0.26
Estimated blood loss (mL)	3	169 180	0	0.55	WMD=-17.14	-29.96~-4.33	0.009
Conversion	5	299 315	36	0.18	WMD=0.58	0.29~1.17	0.13
Dissected lymph node stations	2	195 202	0	0.38	WMD=1.07	0.79~1.36	<0.001
Dissected lymph nodes	2	169 180	0	0.65	WMD=0.98	-0.02~1.97	0.05
Time of chest tube drainage (days)	4	287 298	50	0.11	WMD=-0.34	-0.84~0.15	0.17
Length of hospital stay (days)	5	299 315	72	<0.001	WMD=-0.19	-0.98~0.61	0.65
30-day mortality	3	224 237	0	0	OR=0.20	0.01~4.26	0.30
30-day readmission	5	299 315	38	0.17	OR=0.74	0.36~1.51	0.41
Overall complications	5	299 315	14	0.32	WMD=0.76	0.52~1.11	0.16
Pneumonia	3	232 241	0	0.41	OR=1.65	0.43~6.43	0.47
Pleural effusion	3	232 241	0	1.00	OR=1.04	0.26~4.22	0.96
Atelectasis	2	75 78	19	0.27	OR=1.47	0.28~7.65	0.65
Arrhythmia	3	232 241	0	0.61	OR=1.26	0.37~4.28	0.71
Total cost	2	212 218	99	<0.001	WMD=3103.48	-575.78~6782.74	0.10

**Figure 4:**
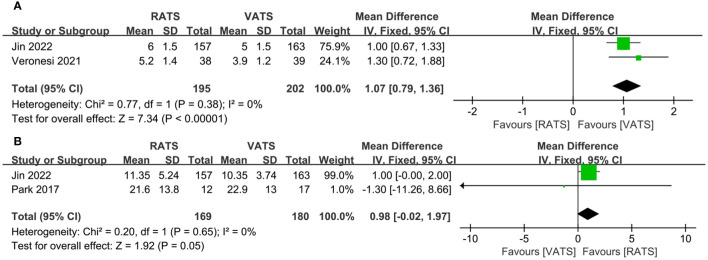
Forest plot of the meta-analysis for pathology details. **(A)** Number of dissected lymph node stations. **(B)** Number of dissected lymph nodes.

The number of dissected lymph nodes stations in RATS group was significantly more than that of VATS groups(WMD = 1.07, 95% CI 0.79 ~ 1.36, P < 0.001) (**Figure 4A**). Two studies reported the number of dissected lymph nodes. Pooled analysis showed that the number of dissected lymph nodes had no significant difference between two groups(WMD = 0.98, 95% CI − 0.02 ~ 1.97, P = 0.05, I^2 =^ 0%) (**Figure 4B**).

**Figure 5:**
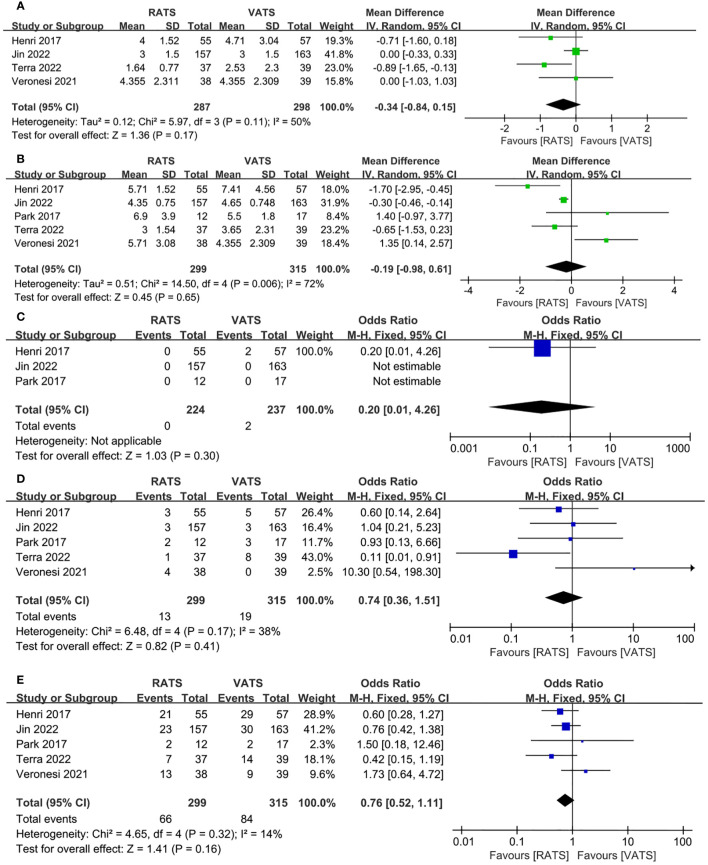
Forest plot of the meta-analysis for postoperative parameters. **(A)** Time of chest tube drainage. **(B)** Length of hospital stay. **(C)** 30-day mortality. **(D)** 30-day readmission. **(E)** Overall postoperative complication.

The time of chest tube drainage had no significant difference between RATS group and VATS group(WMD = −0.34, 95% CI −0.84 ~ −0.15, P =0.17, I^2 =^ 50%) (**Figure 5A**). Pooled analysis showed that the length of hospital stay was not significant different between the RATS and VATS(WMD = −0.19, 95% CI −0.98 ~ 0.61, P =0.65, I^2 =^ 72%) (**Figure 5B**). Pooled analysis of 3 studies showed that no significant difference appeared in the 30-day mortality between RATS and VATS(WMD = 0.20, 95% CI 0.01 ~ 4.26, P =0.30) (**Figure 5C**). 30-day readmission was not significant different between RATS and VATS(OR = 0.74, 95% CI 0.36 ~1.51, P = 0.41, I^2 =^ 38%) (**Figure 5D**). Five studies presented the overall postoperative complication. Pooled analysis showed that there was no significant difference in the rate of overall postoperative complication between the two groups(WMD = 0.76, 95% CI 0.52 ~ 1.11, P =0.16, I^2 =^ 14%) (**Figure 5E**).

**Figure 6:**
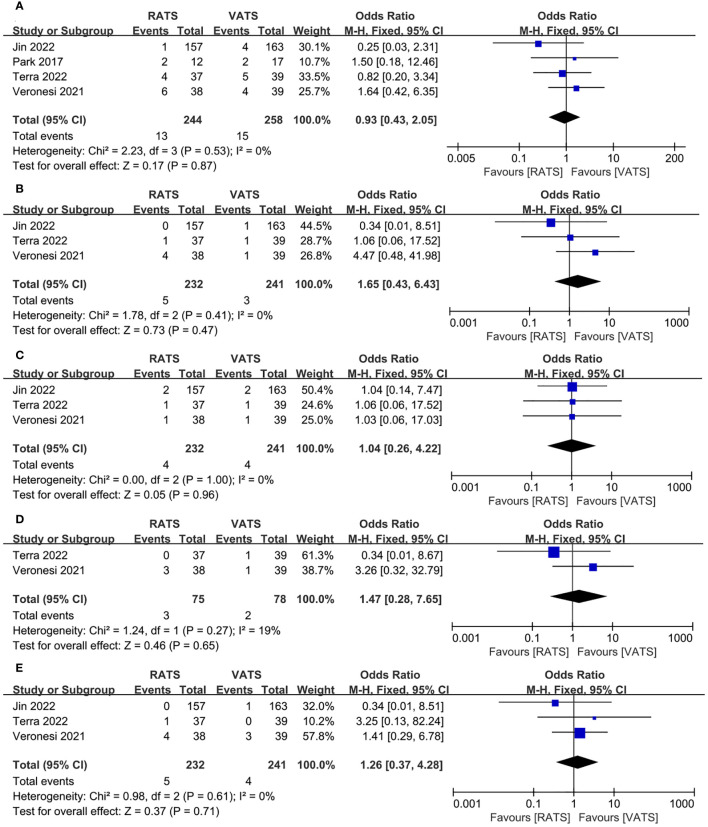
Forest plot of the meta-analysis for common postoperative complication. **(A)** Prolonged air leak. **(B)** Pneumonia. **(C)** Pleural effusion. **(D)** Atelectasis. **(E)** Arrhythmia.

We also analyzed the common complications of RATS and VATS, including of prolonged air leak, pneumonia, pleural effusion, atelectasis, arrhythmia. The results of the analysis showed that RATS and VATS were not statistically significant in prolonged air leak(OR =0.93, 95% CI 0.43 ~2.05, P =0.87, I^2 =^ 0%) (**Figure 6A**), pneumonia(OR =1.65, 95% CI 0.43 ~6.43, P =0.47, I^2 =^ 0%) (**Figure 6B**), pleural effusion(OR =1.04, 95% CI 0.26 ~4.22, P =0.96, I^2 =^ 0%) (**Figure 6C**), atelectasis(OR = 1.47, 95% CI 0.28 ~7.65, P = 0.65, I^2 =^ 19%) (**Figure 6D**) and arrhythmia(OR = 1.26, 95% CI 0.37 ~4.28, P =0.71, I^2 =^ 0%) (**Figure 6E**).

### 3.5 Total cost

**Figure 7:**
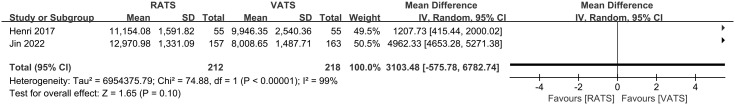
Forest plot of the meta-analysis for total cost.

Only two studies reported total cost for patients, and there was no significant difference between two group (WMD =3103.48, 95% CI −575.78 ~ 6782.74, P =0.1, I^2 =^ 99%) (**Figure 7**).

### 3.6 Upstaging rate

**Figure 8:**
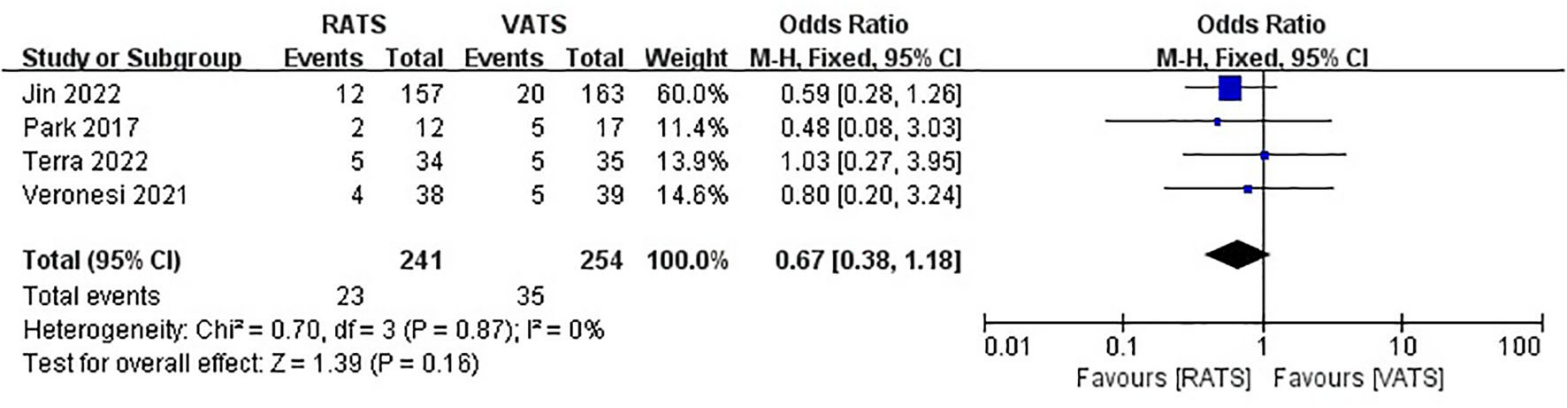
Forest plot of the meta-analysis for upstaging rate.

Four study reported upstaging rate, and there was no significant difference between two group (OR =0.67, 95% CI 0.38 ~ 1.18, P =0.16, I^2 =^ 0%) (**Figure 8**).

### 3.7 Publication of bias

**Figure 9:**
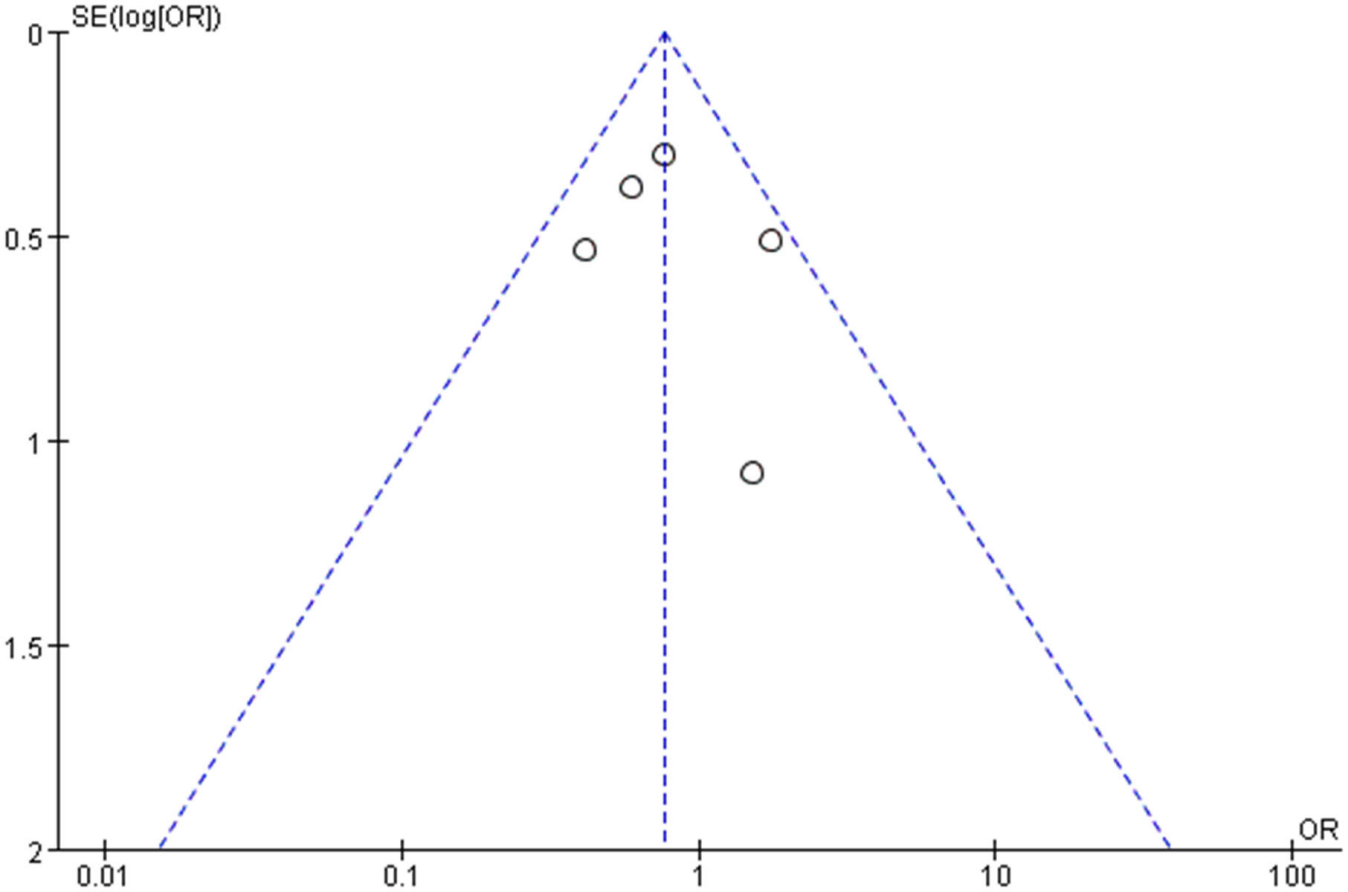
Funnel plot of the overall postoperative complications.

Publication bias of the overall complication was assessed by a funnel plot. No obvious evidence of publication bias was observed in the bilaterally symmetrical funnel plot of overall complication (**Figure 9**).

## 4 Discussion

Radical resection with lymphadenectomy has become the gold standard surgery for NSCLC at an early stage (25, 26). There is an increased enthusiasm for minimally invasive approaches in the management of lung cancer during the past two decades (27). In recent year, as a relatively new platform for minimally invasive lung lobectomy, RATS has been proposed as an alternative to VATS (13). However, previous meta-analysis comparing the clinical outcomes of VATS with RATS has not been sufficient to prove the benefits of RATS (16–19). Due to shortage of high evidence level RATS studies such as randomized controlled trials, these meta-analysis might have a great risk of potential publication and selection bias, influencing the quality of meta-analysis. Therefore, we conducted a high quality meta-analysis including of only randomized controlled trials or prospective cohort studies to compare outcomes of RATS versus VATS in the treatment of lung cancer.

With respect to the operative time, our result showed that there was no statistical difference between RATS and VATS. Though some previous studies reported similar results to ours (18, 28), results of other studies was contrary to our results (6, 29, 30). At the beginning of the learning curve, due to the shortage of experience and knowledge of RATS surgeons who attempts to RATS for lung cancer might need more time to complete the operation. A previous study showed that there was a tendency of gradual shortening in operative time with the increased experience of RATS (31).

Our results showed that the intraoperative blood loss of RATS was less than that of VATS, which was similar to previous study (18). This is likely due to the advantages of more flexible equipment and a three-dimensional magnified vision,which help reveal the complex anatomy around the mediastinum and hilar accurately, resulting in precise manipulation and better control bleeding (17). Regarding the conversion rate, the result in the present study revealed that the conversion rates were not significantly different between two groups.

In terms of lymphadenectomy, our results showed that the number of dissected lymph nodes stations was significantly more in RATS than that in VATS, but there was no significant difference in number of dissected lymph nodes and upstaging rate. Previous studies comparing lymphadenectomy have reported both equivalence (32) and favouring the robotic approach (18, 25, 33, 34). The superior vision and stability is one potential strength of RATS which allows surgeons to perform extensive lymphadenectomy.

Previous study reported shorter drainage and hospital stay of patients in the RATS group than in the RATS group (18), and explained that minimally invasive advantages of RATS contribute to more thorough hemostasis, more delicate operation, less irritation to surrounding tissues such as pleura, which results in less pleural effusion and shorter postoperative hospital stay. However, both time of chest tube drainage and length of hospital stay had no significant difference between two groups in the present study. The small sample size of the included patients may be the main reason.

Kent et al. reported a lower mortality with RATS relative to VATS (35). Liang et al (28) demonstrated the 30-day mortality was lower in RATS group. Another meta-analysis showed that RATS was associated with lower postoperative complication rate (18). The minimally invasive advantages of RATS contributes to less damage and fewer postoperative complications, resulting in lower mortality and readmission. However, regarding complications, 30-day mortality and 30-day readmission, there was no significant difference between two groups in our results. The possible reason is that the surgical outcomes might be affected by other factors, such as the surgeons experience, familiarity with the instrument, and compliance of assistant. Thus, the advantage of RATS need to be confirmed by more prospective randomized controlled studies.

Due to the cost to acquire robot, subsequent maintenance costs and the additional expense of disposable robotic instruments, the total cost of RATS is higher than that of VATS. The high current cost of robotic thoracic surgery may be a worrying limit for popularization and application of RATS. Our results demonstrated a higher total cost patients in RATS group, but this difference was not statistically significant. Since only two of studies included reported results about total cost, the sample size of the included patients was too small to reflect the difference between RATS group and VATS group.

To our knowledge, this is the first meta-analysis including of only randomized controlled trials or prospective cohort studies to compare outcomes of RATS versus VATS in the treatment of lung cancer, which could result in relatively robust conclusion. However, we acknowledge the possible limitations of our study. First of all, only five studies were included duo to our strict inclusion and criteria. The statistical results of partial clinical outcomes were difficult to reflect the difference between the two groups due to the small sample size. Second, we failed to analyse long-term outcomes such as 5-year overall survival because of the short follow-ups of the studies included. Besides, we failed to control confounding factors such as different inclusion criteria, differences on the population and the level of expertise of surgeons involved, which might result in heterogeneity of the studies and bias. Therefore, more clinical outcomes reported by prospective randomized controlled trials are necessary to further confirm the advantage of the RATS.

In conclusion, our study indicated that RATS is a feasible and safe technique that can achieve better surgical efficacy compared with VATS in terms of short-term outcomes. Except of higher total cost, RATS has obvious advantage in lymphadenectomy and control of hemorrhage. However, large sample and long follow-up randomized clinical trials comparing RATS with VATS are still necessary to better demonstrate the advantages of RATS for NSCLC.

## Data availability statement

The original contributions presented in the study are included in the article/**Supplementary Material**. Further inquiries can be directed to the corresponding author.

## Author contributions

WL: Funding acquisition, Project administration, Writing – review and editing. SH: Data curation, Writing – original draft. XH: Data curation, Writing – original draft. ZH: Formal Analysis, Conceptualization, Writing – original draft. RL: Visualization, Software, Writing – original draft.
