# Supplementary material for: Comparison of robot-assisted thoracic surgery versus video-assisted thoracic surgery in the treatment of lung cancer: a systematic review and meta-analysis of prospective studies
Source: Front Oncol. 2023 Oct 30;13:1271709. doi: 10.3389/fonc.2023.1271709 (PMC10646752; doi:10.3389/fonc.2023.1271709)
Supplement: Supplementary file 1 [file Table_1.docx]

**Supplement Tables**

**Supplement Table 1.** The details of the searching record in Medline

| No. | Query | Results |
| --- | --- | --- |
| 1 | ((((((((((((((((("Lung Neoplasms"[Mesh]) OR (Pulmonary Neoplasms[Title/Abstract])) OR (Neoplasms, Lung[Title/Abstract])) OR (Lung Neoplasm[Title/Abstract])) OR (Neoplasm, Lung[Title/Abstract])) OR (Neoplasms, Pulmonary[Title/Abstract])) OR (Neoplasm, Pulmonary[Title/Abstract])) OR (Pulmonary Neoplasm[Title/Abstract])) OR (Lung Cancer[Title/Abstract])) OR (Cancer, Lung[Title/Abstract])) OR (Cancers, Lung[Title/Abstract])) OR (Lung Cancers[Title/Abstract])) OR (Pulmonary Cancer[Title/Abstract])) OR (Cancer, Pulmonary[Title/Abstract])) OR (Cancers, Pulmonary[Title/Abstract])) OR (Pulmonary Cancers[Title/Abstract])) OR (Cancer of the Lung[Title/Abstract])) OR (Cancer of Lung[Title/Abstract]) | 341,852 |
| 2 | ((Robotic[Title/Abstract]) OR (Robot[Title/Abstract])) | 61,034 |
| 3 | ((((((((((((((("Thoracoscopy"[Mesh]) OR (Thoracoscopies[Title/Abstract])) OR (Pleural Endoscopy[Title/Abstract])) OR (Pleuroscopy[Title/Abstract])) OR (Pleuroscopies[Title/Abstract])) OR (Endoscopy, Pleural[Title/Abstract])) OR (Endoscopies, Pleural[Title/Abstract])) OR (Pleural Endoscopies[Title/Abstract])) OR (Surgical Procedures, Thoracoscopic[Title/Abstract])) OR (Surgical Procedure, Thoracoscopic[Title/Abstract])) OR (Thoracoscopic Surgical Procedure[Title/Abstract])) OR (Thoracoscopic Surgery[Title/Abstract])) OR (Thoracoscopic Surgical Procedures[Title/Abstract])) OR (Surgery, Thoracoscopic[Title/Abstract])) OR (Surgeries, Thoracoscopic[Title/Abstract])) OR (Thoracoscopic Surgeries[Title/Abstract]) | 24,819 |
| 4 | ((("Prospective Studies"[Mesh]) OR (Prospective Study[Title/Abstract])) OR (Studies, Prospective[Title/Abstract])) OR (Study, Prospective[Title/Abstract]) | 711,075 |
| 5 | (randomized controlled trial [pt] OR controlled clinical trial [pt] OR randomized [tiab] OR placebo [tiab] OR clinical trials as topic [mesh:noexp] OR randomly [tiab] OR trial [ti]) NOT (animals [mh] NOT humans [mh]) | 1,427,694 |
| 6 | #4 OR #5 | 2,000,348 |
| 7 | #1 AND #2 AND #3 AND #6 | 45 |

**Supplement Table 2.** The details of the searching record in CENTRAL

| No. | Query | Results |
| --- | --- | --- |
| 1 | (Lung Neoplasms OR Pulmonary Neoplasms OR Neoplasms, Lung OR Lung Neoplasm OR Neoplasm, Lung OR Neoplasms, Pulmonary OR Neoplasm, Pulmonary OR Pulmonary Neoplasm OR Lung Cancer OR Cancer, Lung OR Cancers, Lung OR Lung Cancers OR Pulmonary Cancer OR Cancer, Pulmonary OR Cancers, Pulmonary OR Pulmonary Cancers OR Cancer of the Lung OR Cancer of Lung):ab,ti,kw | 33018 |
| 2 | (Robotic OR Robot):ab,ti,kw | 6704 |
| 3 | (Thoracoscopy OR Thoracoscopies OR Pleural Endoscopy OR Pleuroscopy OR Pleuroscopies OR Endoscopy, Pleural OR Endoscopies, Pleural OR Pleural Endoscopies OR Surgical Procedures, Thoracoscopic OR Surgical Procedure, Thoracoscopic OR Thoracoscopic Surgical Procedure OR Thoracoscopic Surgery OR Thoracoscopic Surgical Procedures OR Surgery, Thoracoscopic OR Surgeries, Thoracoscopic OR Thoracoscopic Surgeries):ab,ti,kw | 2254 |
| 4 | (randomized controlled trial OR randomized OR randomised OR randomization OR randomisation OR rct OR randomly OR placebo):ab,ti,kw | 1322723 |
| 5 | (Prospective Studies OR Prospective Study OR Studies, Prospective OR Study, Prospective):ab,ti,kw | 240516 |
| 6 | #4 OR #5 | 1363345 |
| 7 | #1 AND #2 AND #3 AND #6 | 34 |

**Supplement Table 3.** The details of the searching record in Embase

| No. | Query | Results |
| --- | --- | --- |
| 1 | ‘Lung Neoplasms’/exp OR ‘Pulmonary Neoplasms’：ab,ti,kw OR ‘Neoplasms, Lung’：ab,ti,kw OR ‘Lung Neoplasm’：ab,ti,kw OR ‘Neoplasm, Lung’：ab,ti,kw OR ‘Neoplasms, Pulmonary’：ab,ti,kw OR ‘Neoplasm, Pulmonary’：ab,ti,kw OR ‘Pulmonary Neoplasm’：ab,ti,kw OR ‘Lung Cancer’：ab,ti,kw OR ‘Cancer, Lung’：ab,ti,kw OR ‘Cancers, Lung’：ab,ti,kw OR ‘Lung Cancers’：ab,ti,kw OR ‘Pulmonary Cancer’：ab,ti,kw OR ‘Cancer, Pulmonary’：ab,ti,kw OR ‘Cancers, Pulmonary’：ab,ti,kw OR ‘Pulmonary Cancers’：ab,ti,kw OR ‘Cancer of the Lung’：ab,ti,kw OR ‘Cancer of Lung’：ab,ti,kw | 581134 |
| 2 | ‘Robotic’/exp OR ‘Robot’：ab,ti,kw | 97355 |
| 3 | ‘Thoracoscopy’/exp OR ‘Thoracoscopies’：ab,ti,kw OR ‘Pleural Endoscopy’：ab,ti,kw OR ‘Pleuroscopy’：ab,ti,kw OR ‘Pleuroscopies’：ab,ti,kw OR ‘Endoscopy, Pleural’：ab,ti,kw OR ‘Endoscopies, Pleural’：ab,ti,kw OR ‘Pleural Endoscopies’：ab,ti,kw OR ‘Surgical Procedures, Thoracoscopic’：ab,ti,kw OR ‘Surgical Procedure, Thoracoscopic’：ab,ti,kw OR ‘Thoracoscopic Surgical Procedure’：ab,ti,kw OR ‘Thoracoscopic Surgery’：ab,ti,kw OR ‘Thoracoscopic Surgical Procedures’：ab,ti,kw OR ‘Surgery, Thoracoscopic’：ab,ti,kw OR ‘Surgeries, Thoracoscopic’：ab,ti,kw OR ‘Thoracoscopic Surgeries’：ab,ti,kw | 24651 |
| 4 | 'clinical trial'/de OR 'randomized controlled trial'/de OR 'randomization'/de OR 'single blind procedure'/de OR 'double blind procedure'/de OR 'crossover procedure'/de OR 'placebo'/de OR 'prospective study'/de OR 'randomi?ed controlled' NEXT/1 trial* OR rct OR 'randomly allocated' OR 'allocated randomly' OR 'random allocation' OR allocated NEAR/2 random OR single NEXT/1 blind* OR double NEXT/1 blind* OR (treble OR triple) NEAR/1 blind* OR placebo* | 2892432 |
| 5 | ‘Prospective Studies’：ab,ti,kw OR ‘Prospective Study’：ab,ti,kw OR ‘Studies, Prospective’：ab,ti,kw OR ‘Study, Prospective’：ab,ti,kw | 322263 |
| 6 | #4 OR #5 | 2979002 |
| 7 | #1 AND #2 AND #3 AND #6 | 49 |

**Supplement Table 4.** The details of the searching record in Web of Science

| No. | Query | Results |
| --- | --- | --- |
| 1 | TS=(LungNeoplasms OR Pulmonary Neoplasms OR Neoplasms, Lung OR Lung Neoplasm OR Neoplasm, Lung OR Neoplasms, Pulmonary OR Neoplasm, Pulmonary OR Pulmonary Neoplasm OR Lung Cancer OR Cancer, Lung OR Cancers, Lung OR Lung Cancers OR Pulmonary Cancer OR Cancer, Pulmonary OR Cancers, Pulmonary OR Pulmonary Cancers OR Cancer of the Lung OR Cancer of Lung) | 792704 |
| 2 | TS=(Robotic OR Robot) | 238974 |
| 3 | TS=(Thoracoscopy OR thoracoscopic OR Pleural Endoscopy OR Pleuroscopy OR pleuroscopie OR Endoscopy, Pleural OR Endoscopies, Pleural OR Pleural Endoscopies OR Surgical Procedures, Thoracoscopic OR Surgical Procedure, Thoracoscopic OR Thoracoscopic Surgical Procedure OR Thoracoscopic Surgery OR Thoracoscopic Surgical Procedures OR Surgery, Thoracoscopic OR Surgeries, Thoracoscopic OR Thoracoscopic Surgeries) | 28284 |
| 4 | TS= clinical trial* OR TS=research design OR TS=comparative stud* OR TS=evaluation stud* OR TS=controlled trial* OR TS=follow-up stud* OR TS=prospective stud* OR TS=random* OR TS=placebo* OR TS=(single blind*) OR TS=(double blind*) | 11214105 |
| 5 | TS=(Prospective Studies OR Prospective Study OR Studies, Prospective OR Study, Prospective) | 1047867 |
| 6 | #4 OR #5 | 11214105 |
| 7 | #1 AND #2 AND #3 AND #6 | 218 |
